# Osteosarcoma Originating From a Malignant Phyllodes Tumor: A Case Report of a Rare Malignancy

**DOI:** 10.7759/cureus.37737

**Published:** 2023-04-17

**Authors:** Nishi Jha, Monika Maharjan, Rajesh Rimal, Farhanul Huda, Ashok Singh

**Affiliations:** 1 Department of Pathology and Laboratory Medicine, All India Institute of Medical Sciences, Rishikesh, IND; 2 Department of Radiology, All India Institute of Medical Sciences, Rishikesh, IND; 3 Department of General Surgery, All India Institute of Medical Sciences, Rishikesh, IND

**Keywords:** breast, heterologous, osteosarcoma, phyllodes, malignant

## Abstract

Fibroepithelial tumors are common in the breast; however, the frequency of malignancy is much less as compared to the epithelial counterpart. Malignant phyllodes are infrequent, and the proportion undergoing heterologous differentiation is very rare. Extensive sampling and astute examination are of utmost importance so as not to miss this lesion. The prognosis of these tumors is worse compared to cases where no heterologous transformation is seen.

## Introduction

Phyllodes tumors (PTs) are uncommon fibroepithelial lesions of the breast that account for less than 1% of mammary gland malignancies. They can be benign, borderline, or malignant [1]. The majority of them are benign, and only a small percentage, between 10% and 30%, of phyllodes undergo malignant transformation [2]. Around 80% of malignant phyllodes are confined to the breast at the time of initial presentation, followed by 8.2% of cases with regional disease, lymph node involvement, or direct extension into neighboring tissue and 1.5% of cases with metastatic disease [2]. The presence of heterologous sarcomatous elements within the tumor, such as osteosarcoma, liposarcoma, and chondrosarcoma, directly subjects the phyllodes as malignant regardless of other histopathologic features that are characterized by marked stromal cellularity, nuclear atypia, stromal overgrowth, more than 10 mitoses per high-power field, and infiltrative tumor margins [3]. Although ulceration and adhesion to the chest wall are unusual occurrences, when these tumors are malignant, their sizes range from 4 to 7 cm and patients frequently present with rapid tumor growth [4]. Various imaging modalities and pathologic characteristics play a significant role in distinguishing breast osteosarcoma from other benign and malignant disorders. Malignant phyllodes with osteosarcomatous transformation are highly rare and commonly confused with benign big calcifications [5]. We present a case of a 32-year-old female with a malignant phyllodes tumor (PT) in her left breast with osteosarcomatous transformation.

## Case presentation

**Figure 1:**
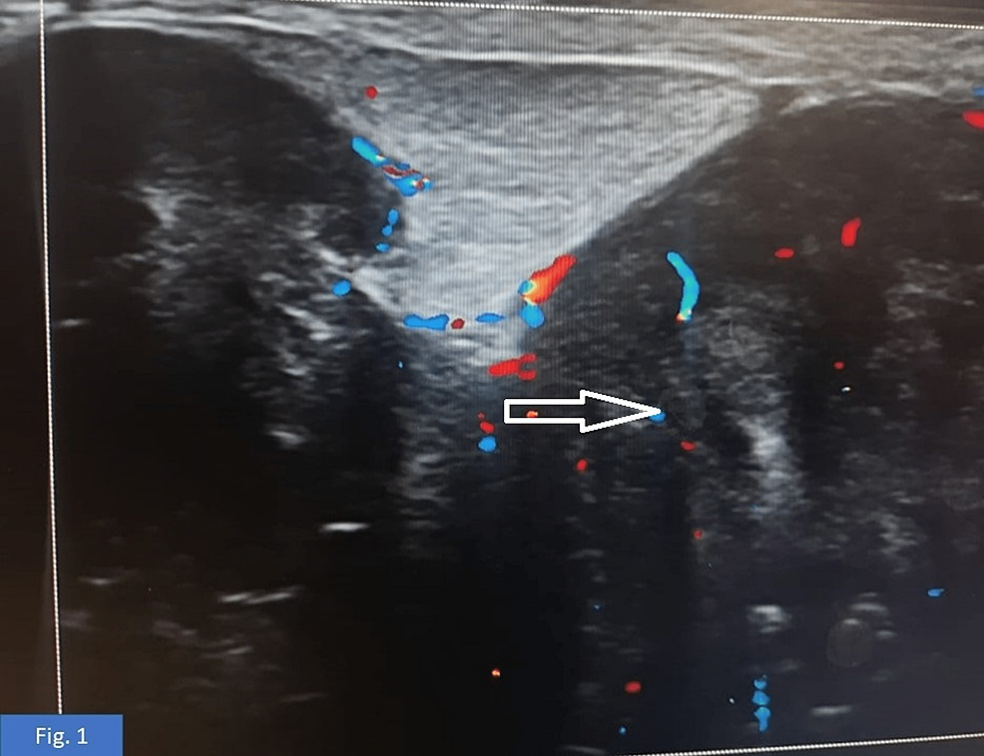
Large, irregularly shaped hypoechoic lesion with partially circumscribed and partially microlobulated margins in the upper outer and lower outer quadrant from 1 to 5 o’clock position.

**Figure 2:**
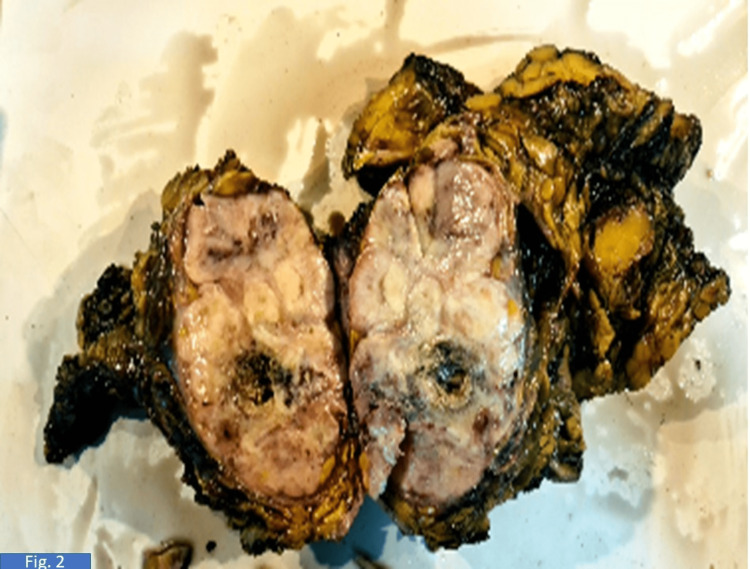
On gross examination, a gray-white lobulated tumor occupying almost the whole of the breast is identified.

A 32-year-old female presented with swelling in the left breast, gradual in onset, progressive in nature, associated with heaviness, and not associated with pain, nipple discharge, or nipple retraction. There was no history of trauma and no history of yellowish discoloration of the eyes or bone pains, as well as no significant past history or family history. General examination and laboratory parameters were within normal limits. The left breast measured 10 × 12 cm with a hard lump present in the lower outer quadrant, not fixed to the skin or muscle. A firm, mobile lymph node measuring 1 × 1 cm was palpated in the left axilla. The right breast and right axilla were within normal limits. The rest of the systemic examination was normal. Ultrasonography of the left breast revealed a predominantly glandular breast parenchyma with a large irregularly shaped hypoechoic lesion with partially circumscribed (Figure 1) and partially microlobulated margins seen in the upper outer and lower outer quadrant measuring 104.7 × 68.9 mm, and there was internal vascularity upon application of color Doppler scanning. Findings were suggestive of Breast Imaging Reporting and Data System (BI-RADS) 4b. A biopsy was done from the representative site. Initially, we received linear cores on which a provisional diagnosis of borderline phyllodes of the left breast was made, following which the patient underwent a left-sided total mastectomy. We received a left-sided total mastectomy specimen (Figure 2).

**Figure 3:**
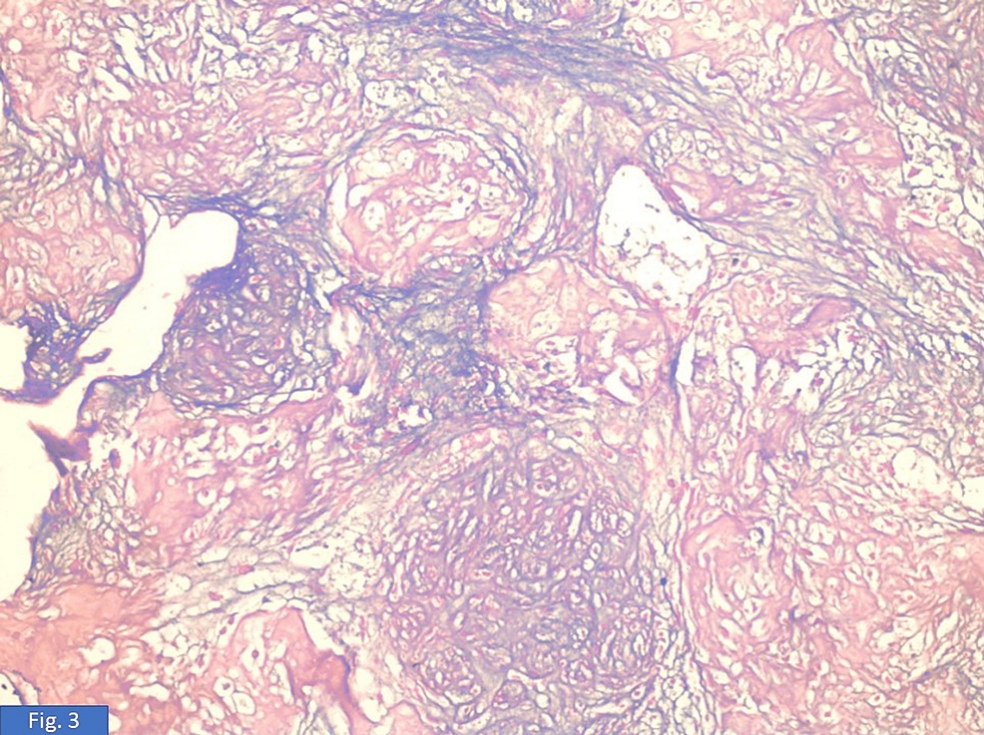
Section shows multiple lobules of fibroepithelial tumor with areas of bone formation (H&E, 100×). H&E: hematoxylin and eosin

**Figure 4:**
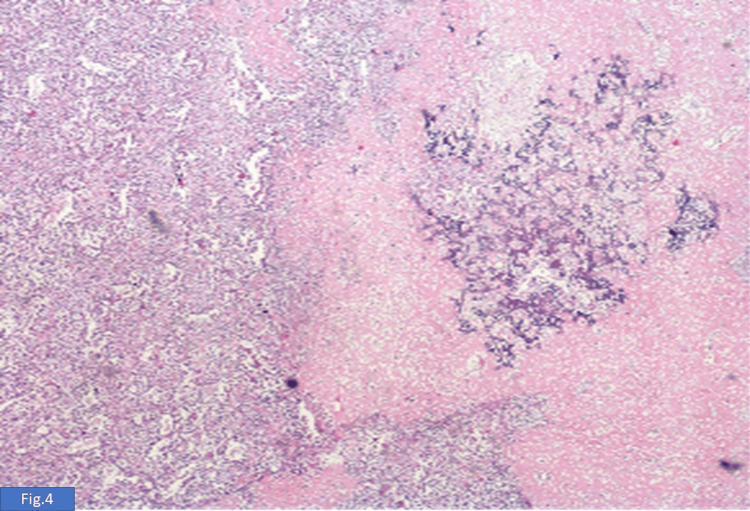
Section shows another area with fungal hyphae-like bone formation. The left side of the micrograph shows a malignant phyllodes tumor (H&E, 100×). H&E: hematoxylin and eosin

**Figure 5:**
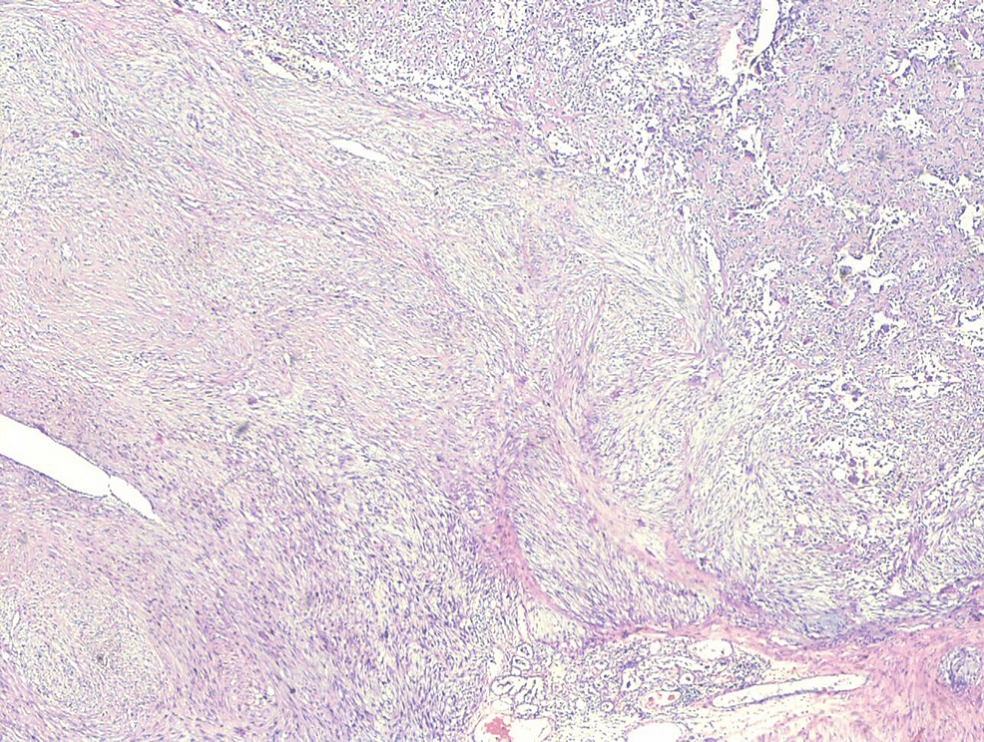
Section shows malignant phyllodes tumor on the left side of the image and osteosarcoma on the right side (H&E, 40×). H&E: hematoxylin and eosin

**Figure 6:**
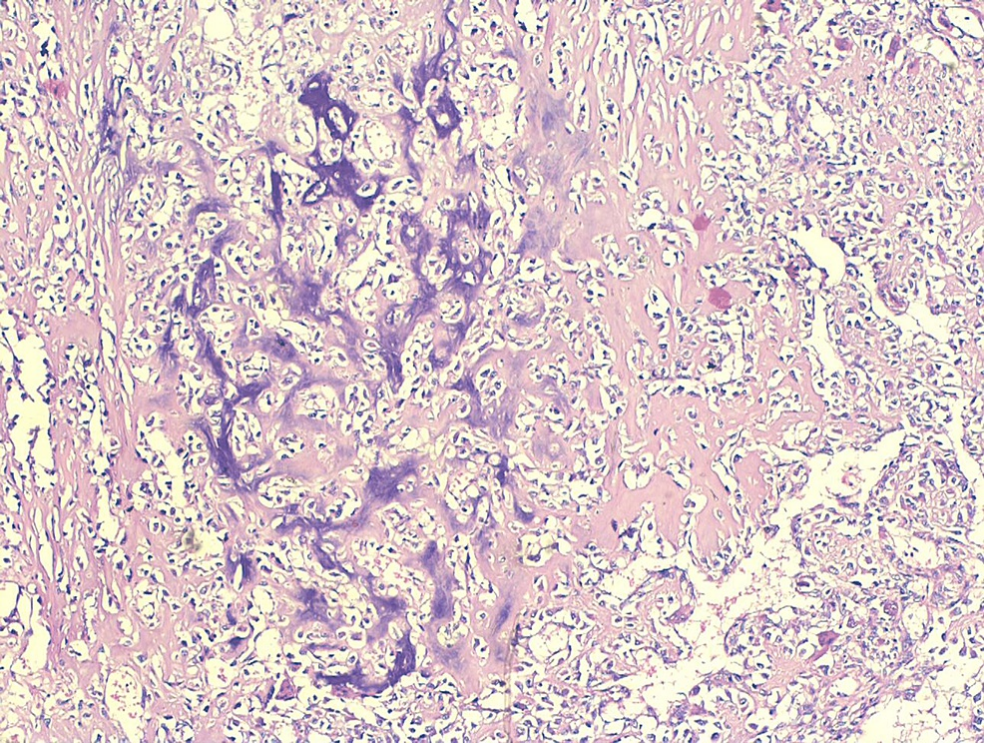
Section shows typical areas showing osteoblastic activity with atypical osteoblastic lining, cells showing high N:C ratio, and hyperchromasia and marked nuclear pleomorphism along with lace-like osteoid formation (H&E, 100×). H&E: hematoxylin and eosin

On gross examination, a 10 × 10 cm hard, variegated mass was identified in the upper outer quadrant exhibiting circumscribed borders. The overlying skin appeared unremarkable. On paraffin section examination, a diagnosis of osteosarcoma (30%) with an osteoblastic component originating from malignant phyllodes (70%) was seen (Figure 3). The tumor was mainly composed of osteosarcomatous areas, and areas of cartilaginous differentiation were also noted (Figure 4 and Figure 5), with well-differentiated trabeculae (Figure 6) in the central area, which were connected to each other. Tumor cells showed obvious atypia (Figure 6), increased cellularity, and focal infiltrative growth pattern, and the TNM stage given was pT4N0Mx. The course during the hospital stay was uneventful. The patient was hemodynamically stable at discharge.

## Discussion

Phyllodes tumor is a rare breast tumor comprising about 0.3%-0.9% of all breast tumors with a predilection for women in their late fifth to sixth decade of life, and malignant phyllodes tumors account for an even smaller cohort (6.5%-27% of all phyllodes tumors). Moreover, it occurs in a population comparable to benign tumors, with rare occurrences in the young population [6]. Phyllodes tumors are uncommon mammary gland cancer, the prognosis of which is highly related to tumor size greater than 5 cm and the histological osteosarcomatous subtype, which increases mortality by 33% [1]. These are periductal stroma-derived, rapidly growing tumors that have both stromal and epithelial components [7]. The risk factors for local recurrence include large tumor size, stromal overgrowth, high mitotic count, nuclear atypia, pleomorphism, and insufficient surgical margins [8,9]. According to Silver et al. [10], phyllodes with osteosarcomatous components are potentially aggressive neoplasms that can spread to the lung, brain, contralateral breast, and other distant organs, as well as cause tumor-related death. Only a few dozen examples of osteosarcomatous differentiation of a malignant phyllodes tumor have been documented to date, accounting for 1.3% of phyllodes tumors in the breast [10]. Certain malignant phyllodes tumors have the ability to induce or spontaneously undergo differentiation to other lineages and exhibit the properties of mesenchymal stem cells, which results in heterologous differentiation [10]. This tumor needs to be distinguished from metaplastic cancer, and according to certain data, malignant phyllodes tumors with heterologous osteosarcomatous differentiation are more aggressive, but their risk of metastasis is substantially lower than that of conventional osteosarcomas in general [11]. Some evidence suggests that malignant phyllodes tumors with osteosarcomatous differentiation are more aggressive, but compared with osteosarcomas in general, they have a much lower risk of metastasis [12]. This tumor needs to be distinguished from metaplastic cancer, and to diagnose it, the tumor epithelial component must be confirmed on immunohistochemistry. The majority of metaplastic carcinoma exhibit immunopositivity for p63 and cytokeratin (CK) [13].

## Conclusions

Malignant phyllodes tumors are infrequent, and osteosarcomatous differentiation is still rarer. A thorough sampling and prior knowledge of the condition are essential for not missing this entity. Early detection and accurate characterization are essential as it has a poorer prognosis.
